# Comparative Study of Prebiotics for Infants Using a Fecal Culture System: Insights into Responders and Non-Responders

**DOI:** 10.3390/nu16193347

**Published:** 2024-10-02

**Authors:** Shijir (Xijier) Mingat, Tatsuya Ehara, Hirohiko Nakamura, Kazuhiro Miyaji

**Affiliations:** Health Care & Nutritional Science Institute, R&D Division, Morinaga Milk Industry Co., Ltd., 5-1-83, Higashihara, Zama 252-8583, Japan; t-ehara@morinagamilk.co.jp (T.E.); hi_nakam@morinagamilk.co.jp (H.N.); k_miyazi@morinagamilk.co.jp (K.M.)

**Keywords:** infant formula, bifidobacteria, human-residential bifidobacteria, prebiotics, non-digestible oligosaccharide, non-responder

## Abstract

Background: The gut microbiota of breast-fed infants is dominated by infant-type human-residential bifidobacteria (HRB) that contribute to infant health; thus, it is crucial to develop infant formulas that promote the establishment of a gut microbiota enriched with infant-type HRB, closely resembling that of breastfed infants. Methods: We compared various non-digestible prebiotic oligosaccharides and their combinations using a fecal culture system to explore which candidates could promote the growth of all infant-type HRB and rarely yield non-responders. The analysis included lactulose (LAC), raffinose (RAF), galactooligosaccharides (GOS), and short- and long-chain fructooligosaccharides. Fecal samples were collected from seven infants aged 1.5–10.2 months and cultured with each oligosaccharide individually or their combinations. Results: No single oligosaccharide effectively promoted the growth of all infant-type HRB, although GOS promoted the growth of HRB other than *Bifidobacterium longum* subsp. *longum*. Only the LAC/RAF/GOS group evenly and effectively promoted the growth of all infant-type HRB. Accordingly, acetate production was higher in fecal cultures supplemented with GOS or LAC/RAF/GOS than in the other cultures, suggesting that it is a superior combination for all infant-type HRB and rarely yields non-responders. Conclusions: This study can aid in developing infant formulas that help align the gut microbiota of formula-fed infants with that of breastfed infants.

## 1. Introduction

Bifidobacteria are the predominant bacteria in the gut microbiota of infants [1] and have different ecological adaptations among species. The species hosted mainly by humans are grouped as human-residential bifidobacteria (HRB) [2]. Among them, *Bifidobacterium breve*, *Bifidobacterium longum* subsp. *infantis*, *Bifidobacterium longum* subsp. *longum*, and *Bifidobacterium bifidum* are classified as infant-type HRB as they prevalently reside in the infant intestine [2]. These HRB have several potential health benefits for infants, including the production of certain nutrients [3], regulation of intestinal function [4], prevention of infections [5], regulation of immune system development [6], modulation of gut immune and endocrine functions [7], and improvement in vaccine response [8]. These beneficial effects seem to be mediated, at least partially, by certain metabolites, such as acetate and tryptophan metabolites produced by bifidobacteria [9,10].

Breastfeeding is strongly recommended not only because of the many functional bioactive components in breast milk, such as defense molecules and growth factors [11,12], but also due to its positive impact on gut microbiota. Bifidobacteria in the gut microbiota of formula-fed infants are often less abundant than in the gut microbiota of exclusively breastfed infants [13]. Therefore, one of the key developmental goals of infant formulas is to mimic the bifidogenic properties of breast milk, providing infants who cannot be breastfed with a bifidobacteria-enriched gut microbiota similar to that of a breastfed infant.

Human milk oligosaccharides (HMOs) are putative primary factors in the formation of bifidobacteria-enriched gut microbiota in breastfed infants [14]. They are the third largest structurally diversified component of human breast milk, with more than 200 types reported [15]. HMOs are selectively utilized by infant-type HRB as the sole carbon source via species-specific pathways. They act as prebiotics by promoting their proliferation and aid in the development of bifidobacteria-enriched gut microbiota [16,17]. Recently, some HMOs have been industrially produced and added to infant formula [18,19,20], but the reproduction of diverse whole HMO complexes remains a significant challenge. Commercially available non-digestible prebiotic oligosaccharides are useful alternatives for increasing bifidobacteria. Many types of prebiotic oligosaccharides are used in infant formula, including lactulose (LAC) [21,22], raffinose (RAF) [23], galactooligosaccharides (GOS) [24], short-chain fructooligosaccharides (scFOS) [25], and long-chain fructooligosaccharides (lcFOS) [26], either alone [21,22,23,24,25,26,27] or in combination [27,28,29].

Because structurally different oligosaccharides require distinct metabolic pathways to be utilized [30], a certain prebiotic oligosaccharides may yield responders and non-responders, depending on whether the targeted bacteria in the individual gut microbiota possess related gene sets [31]. In the case of HRB, gene sets of transporters and/or enzymes for carbohydrate utilization are diverse among or even within species [30]. This may make it challenging for a single prebiotic oligosaccharide to promote the growth of all infant-type HRB at the species or strain level.

In this context, the structural diversity of whole HMOs in breast milk seems to provide an advantage for the survival of infant-type HRB in terms of the choice of energy source, which could lead to a low yield of non-responders to HMOs. We previously examined the bifidogenic effect of structurally different combinations of three oligosaccharides (LAC/RAF/GOS) in comparison with LAC alone, LAC/RAF, and LAC/GOS to clarify the advantage of the prebiotic mixture’s structural diversity [29]. We revealed a synergistic bifidogenic effect of LAC/RAF/GOS in an in vitro mixed culture model of artificial infant microbiota [29], as well as additional physiological benefits of LAC/RAF/GOS in the modulation of gut immune and endocrine functions in a neonatal mouse model [7]. However, it remained unclear whether the combination of the prebiotic oligosaccharides shows effective bifidogenic effects on all infant-type HRB in the complex microbiota and is less likely to yield non-responders.

In the present study, we investigated commercially available oligosaccharides and their combinations to determine which would support the full growth of all infant-type HRB, i.e., we explored which oligosaccharides rarely yield non-responders. We conducted a well-described fecal batch culture under pH-controlled and anaerobic conditions as a model of infant gut microbiota to compare their growth with various oligosaccharides and their combinations. The results showed that LAC/RAF/GOS promoted all infant-type HRB in all tested feces, indicating that this combination could be a useful prebiotic mixture for infants, rarely yielding non-responders.

## 2. Materials and Methods

### 2.1. Oligosaccharides

The oligosaccharides used in our analyses included LAC (99.9%; Morinaga Milk Industry Co., Ltd., Tokyo, Japan), RAF (>98%; Nippon Beet Sugar Manufacturing Co., Ltd., Tokyo, Japan), short-chain fructooligosaccharides (scFOS) (>93.0%; FUJIFILM Wako Pure Chemical Corporation Co., Ltd., Tokyo, Japan; mixture of 1-kestose (trisaccharide), nystose (tetrasaccharide), and fructofuranosyl nystose (pentasaccharide)), long-chain fructooligosaccharides (lcFOS) (86.2%; TEIJIN Co., Ltd., Tokyo, Japan; the average chain length ranges from 8 to 13 fructose units), and GOS (composed of approximately 14% tetrasaccharides, 82% trisaccharides, and 2% disaccharides). GOS were obtained by the purification of Oligomate 55N (Yakult Honsha Co., Ltd., Tokyo, Japan) using gel filtration column chromatography, as previously reported [32].

### 2.2. Fecal Samples

This study was approved by the Japan Conference of Clinical Research (protocol code 101-034), and written informed consent was obtained from the parents. Seven healthy Japanese infants aged 1.5–10.2 months were recruited, and one fecal sample was collected from each subject at the time point shown in Table 1. All the infants were delivered vaginally and breastfed. Among them, four infants were partially breastfed, and three had already been introduced to solid foods. Immediately after collection, the fecal samples were maintained under anaerobic conditions using AnaeroPack (Mitsubishi Gas Chemical Co., Ltd., Tokyo, Japan) at <10 °C, and diluted within 8 h. The fecal samples were diluted 10 times with saline (Otsuka Pharmaceutical Factory, Inc., Tokushima, Japan), vortexed, and stored at −80 °C until the experiment [32,33].

### 2.3. In Vitro Fecal Fermentation

In vitro fecal fermentation was performed using a pH-controlled multichannel jar fermenter (Bio Jr. 8; ABLE, Tokyo, Japan), as previously described [33]. In brief, 100 µL of a 10-fold diluted fecal sample (containing 10 mg of feces) was inoculated into 100 mL of yeast extract, casitone, and fatty acids (YCFA) medium. The medium was cultured at a constant temperature of 37 °C under anaerobic conditions (100% CO_2_) with agitation at 80 rpm for 24 h, with a pH of 7.0 and minimum pH of 5.5, as the pH in the colon of a healthy infant typically does not fall below 5.5 [32]. When the prebiotics were used in combination, their weight ratios were 1/1/1 (LAC/RAF/GOS), 1/1 (RAF/GOS, LAC/GOS, and LAC/RAF), and 1/9 (lcFOS/GOS). The total oligosaccharide concentration in each culture medium was 1.0% (*w*/*v*). Culture media were collected before (at 0 h) and after (at 24 h) cultivation.

### 2.4. DNA Extraction

For DNA extraction, the bead-beating method was used as described previously [33]. Samples of the fecal culture medium (1 mL) were centrifuged at 4 °C and 8000× *g* for 3 min to obtain the cell pellets. Lysis buffer (No. 10, Kurabo Industries Ltd., Osaka, Japan; 300 μL) and 0.3 g of 0.1 mm glass beads were added to either the pellet or 20–30 mg fecal sample prior to culture, after which the mixture was disrupted using Fast prep-24 5G cell disrupter (Funakoshi Co., Ltd., Tokyo, Japan), at room temperature for 60 s at speed 5.0. It was subsequently kept on ice for 5 min. This step was repeated five times. The homogenized sample was centrifuged at 4 °C and 13000× *g* for 5 min, and 0.2 mL of supernatant was collected. Protease K (FUJIFILM Wako Pure Chemical Corporation Co., Ltd., Tokyo, Japan) was dissolved in 150 μL (final titer of 9 unit/mL) of No. 2 reagent (Kurabo Industries Ltd., Osaka, Japan) and added to the supernatant. No. 10 reagent (300 μL) was also added to the supernatant. The supernatant was then processed using a GenePrep Star PI-480 automatic DNA extraction machine (Kurabo Industries Ltd., Osaka, Japan) to obtain the extracted DNA, which was then subjected to real-time PCR analysis.

### 2.5. Quantification of Bacterial Cell Numbers

For the quantification of bacterial cell numbers, real-time PCR was performed using an ABI PRISM 7500 Fast Real-Time PCR System (Thermo Fisher Scientific, Waltham, MA, USA) and TBGreen^®^ Premix Ex Taq^TM^ Tli RNaseH Plus (TaKaRa Bio Inc., Shiga, Japan). The primers used are listed in Supplemental Table S1 [34,35,36,37]. PCR was performed under the following conditions: initial hold stage at 95 °C for 20 s, followed by 40 cycles at 95 °C for 5 s, 55 °C for 20 s, and 72 °C for 30 s. Cell numbers in culture media at 0 h and 24 h cultivation were calculated using standard curves. The log-fold changes in cell numbers in the culture media during 24 h of cultivation were determined for *Bifidobacterium*, *B. breve*, *B. longum* subsp. *longum*, *B. longum* subsp. *infantis*, and *B. bifidum* and used for statistical analyses and heat-map drawing.

### 2.6. SCFAs and Lactate Analysis

SCFAs (acetate, propionate, and butyrate) and lactate were measured using a YMC Fatty Acid Analysis Kit (YMC Co., Ltd., Kyoto, Japan) with 2-nitrophenylhydrazine (2-NPH) as the derivatizing agent, with certain modifications to the kit protocol. The culture supernatant was obtained by centrifugation at 4 °C and 8000× *g* for 10 min, filtered through a 0.22 μm filter (PVDF membrane, Merck Millipore Ltd., Cork, Ireland), diluted to the appropriate concentration with Milli-Q, and used as a sample. Furthermore, 10 μL of 2.5 mM ethylbutyric acid (FUJIFILM Wako Pure Chemical Corp., Osaka, Japan) was added to 10 μL of the sample as an internal standard, followed by addition of 40 μL of kit reagents A and B. The obtained mixture was heated at 60 °C for 20 min, followed by addition of reagent C, thorough mixing, addition of 800 μL of reagent D and 1000 μL of n-hexane (FUJIFILM Wako Pure Chemical Corp., Osaka, Japan), and thorough mixing. The mixture was centrifuged at 600 rpm for 30 s to remove the upper layer (long-chain fatty acids), followed by addition of 1000 μL of hexane, mixing, centrifugation at 600 rpm for 30 s, and removal of the upper layer. After the addition of 1000 μL of diethyl ether (FUJIFILM Wako Pure Chemical Corp., Osaka, Japan) to the lower layer, the mixture was mixed by rotating for 30 min and centrifuged at 3000 rpm for 3 min. The upper layer (400 μL) was collected and dried by spraying with N_2_. The obtained product was dissolved with 200 μL of methanol (FUJIFILM Wako Pure Chemical Corp., Osaka, Japan), filtered, and used as an HPLC sample. Chromatographic separation was performed using the modified YMC-Pack FA method on a Waters HPLC system (Waters Corporation, Milford, MA, USA). A YMC-Pack FA column (250 × 6.0 mm I. D. YMC Co., Ltd., Kyoto, Japan) was used for chromatographic separation. The eluent was a mixture of acetonitrile (KOKUSAN CHEMICAL Co., Ltd., Tokyo, Japan), methanol, and ultrapure water (KOKUSAN CHEMICAL Co., Ltd., Tokyo, Japan) at a ratio of 5:16:79. The pH was adjusted to 4.5 using 0.1% trifluoroacetic acid (KOKUSAN CHEMICAL Co., Ltd., Tokyo, Japan). The elution program was designed to gradually increase the acetonitrile concentration from 5% to 30% over a period of 5–55 min. This was followed by maintaining a constant flow rate for an additional 10 min. Subsequently, the acetonitrile concentration was decreased from 30% to 5% over 10 min. Finally, the column was re-equilibrated for 5 min. The experiment was conducted with a flow rate of 1.0 mL/min, and the column temperature was maintained at 50 °C. Detection was performed using UV light at 400 nm, with a sensitivity of 0.005 absorbance units full scale (AUFS). The quantity of SCFAs and lactate produced during cultivation was calculated by subtracting the concentration at 0 h from that at 24 h.

### 2.7. Statistical Analysis

Statistical analyses were performed using JMP software version 13 (SAS Institute, Cary, NC, USA). Analysis of covariance (ANCOVA) was applied to the log-fold changes in the cell numbers of *Bifidobacterium*, *B. breve*, *B. longum* subsp. *longum*, *B. longum* subsp. *infantis*, and *B. bifidum* to compare the least squares means (LSMeans) and rank the experimental groups. Difference in responsiveness between the subjects was set as a covariate. Heat maps of the log-fold changes in *Bifidobacterium*, *B. breve*, *B. longum* subsp. *longum*, *B. longum* subsp. *infantis*, and *B. bifidum* were created as follows: experimental groups were arranged from top to bottom and subjects from left to right of the panel, according to the magnitude of the effect on the targeted bifidobacteria based on the ANCOVA LSMeans test. Because this experiment was exploratory, that is, exploring a possible candidate for a superior oligosaccharide or combination of oligosaccharides, multiplicity was not considered. Student’s *t*-test was used to explore the statistical significance between the experimental groups. Correlations between the total bifidobacterial count and concentrations of acetate or lactate were analyzed using Pearson’s correlation analysis.

## 3. Results

### 3.1. Characteristics of the Subjects

Feces were collected from seven infants aged 1.5–10.2 months. Three infants were exclusively breastfed, while the others were partially breastfed. Three of the infants had already consumed solid food. As previously reported [38], numbers and combinations of bifidobacterial species in the gut microbiota were diversified among these infants. Two infants possessed only *B. breve*; one possessed *B. longum* subsp. *longum* and *B. longum* subsp. *infantis*; two possessed *B. breve*, *B. longum* subsp. *longum*, and *B. longum* subsp. *infantis*; and two possessed all four infant-type HRB, i.e., *B. breve*, *B. longum* subsp. *longum*, *B. longum* subsp. *infantis*, and *B. bifidum*. These characteristics are summarized in Table 1 and Table 2.

### 3.2. Effects of Various Prebiotic Oligosaccharides and Their Combinations on the Growth of Individual Infant-Type Bifidobacteria

We selected LAC, RAF, GOS, scFOS, and lcFOS—well-characterized prebiotic oligosaccharides in infant formulas worldwide—to investigate whether any single oligosaccharide or combination of oligosaccharides can effectively promote the growth of all infant-type bifidobacteria across the various infant gut microbiota tested. Experimental groups were set up to assess the single oligosaccharides and their combinations, as summarized in Table 3. Each fecal sample was added to the test medium containing oligosaccharide(s), as shown in Table 3, and cultured for 24 h. After 24 h of cultivation, the total bacteria were slightly higher in the scFOS group than in the GOS and lcFOS/GOS groups and comparable among the other groups (Supplemental Figure S1).

**Figure 1:**
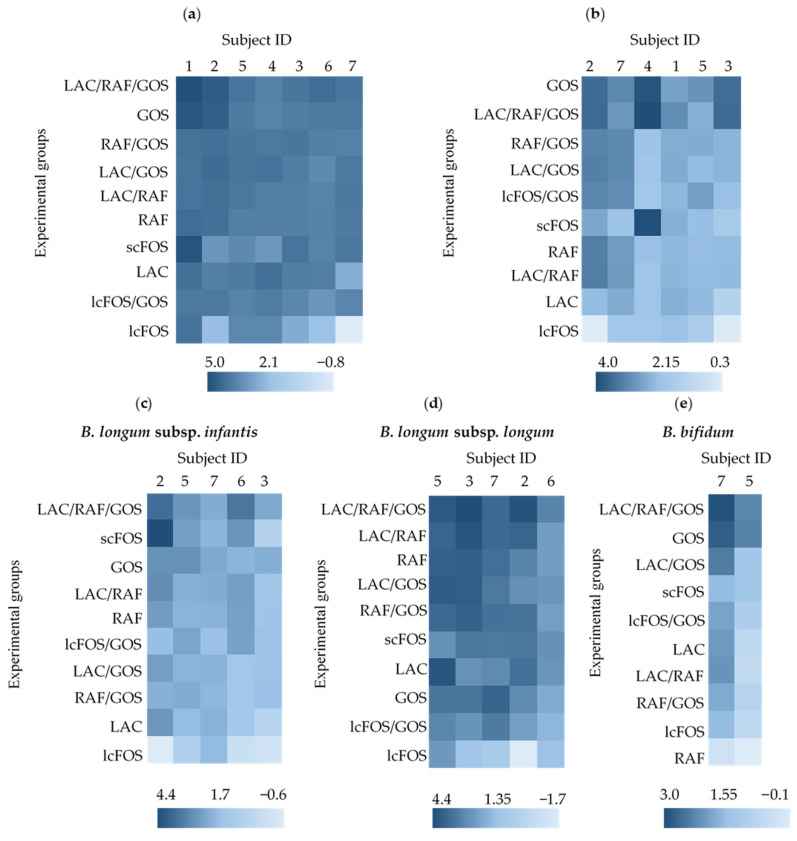
Heat-mapping and ranking of the prebiotic effects of oligosaccharide (s) on infant-type HRB. The prebiotic effects of oligosaccharide (s) on the targeted bifidobacteria were heat-mapped with corresponding log-fold changes in cell numbers during cultivation. Ranking was performed as described in the Section 2. Experimental groups are arranged from top to bottom of the panel and subjects from left to right of the panel, according to the size of the effect on the targeted bifidobacteria, based on the ANCOVA LSMeans test. Overall, the group arranged at the top was the most effective. (**a**): *Bifidobacterium*; (**b**): *Bifidobacterium breve*; (**c**): *Bifidobacterium longum* subsp. *infantis*; (**d**): *Bifidobacterium longum* subsp. *longum*; (**e**): *Bifidobacterium bifidum*.

The growth of total bifidobacteria in each infant fecal sample was equally promoted by all single oligosaccharides and their combinations, except for lcFOS. In the lcFOS group, total bifidobacteria did not increase greatly in certain infant fecal samples, such as those from subjects 2 and 7 (Figure 1a). Consistent with this, the average total bifidobacterial count was significantly lower in the lcFOS group than in the other groups (Supplemental Figure S2a). The growth of *B. breve* was effectively promoted in all fecal samples when GOS or LAC/RAF/GOS was added to the medium, but not when other oligosaccharides were added (Figure 1b). For example, *B. breve* in subject 4 did not respond well to oligosaccharides other than GOS, LAC/RAF/GOS, and scFOS, whereas *B. breve* in subject 3 did not respond well to oligosaccharides other than GOS and LAC/RAF/GOS (Figure 1b). The overall growth rate of *B. longum* subsp. *infantis* in the fecal samples was lower than those of the other species (Figure 1c). Among the oligosaccharides and their combinations, LAC/RAF/GOS promoted the growth of *B. longum* subsp. *infantis* in each fecal samples most evenly, and scFOS, GOS, LAC/RAF, and RAF showed comparable growth promotion effects on *B. longum* subsp. *infantis* in each fecal sample (Figure 1c). Among the infant-type HRB, *B. longum* subsp. *longum* appeared to be the most responsive to the tested oligosaccharide(s), except for GOS and lcFOS (Figure 1d). Still, the growth-promoting effect of LAC/RAF/GOS was ranked the highest among the groups, and it seemed to promote the growth of *B. longum* subsp. *longum* in each fecal sample (Figure 1d). Only two infants possessed *B. bifidum* in their gut microbiota; therefore, statistical significance could not be determined. *B. bifidum* in both of these fecal samples showed large difference in response to each single oligosaccharide or their combination. Specifically, the growth of *B. bifidum* in subject 7 was promoted effectively by LAC/RAF/GOS and GOS, and slightly by LAC/GOS, lcFOS/GOS, LAC, LAC/RAF, RAF/GOS, and scFOS, while the growth of *B. bifidum* in subject 5 was promoted effectively by LAC/RAF/GOS and GOS but was not promoted by other oligosaccharide(s) (Figure 1e). As a single oligosaccharide, GOS may be effective for the growth of *B. breve*, *B. longum* subsp. *infantis*, and *B. bifidum*, but not for the growth of *B. longum* subsp. *longum*. *B. longum* subsp. *longum* was significantly increased by RAF among the single oligosaccharide groups (Figure 1b–e). Focusing on combination of oligosaccharides, LAC/RAF/GOS could be effective for the growth of all infant-type HRB among the combination of oligosaccharide groups (Figure 1b–e).

### 3.3. Effects of Prebiotic Oligosaccharides and Their Combinations on SCFAs and Lactate Production

**Figure 2:**
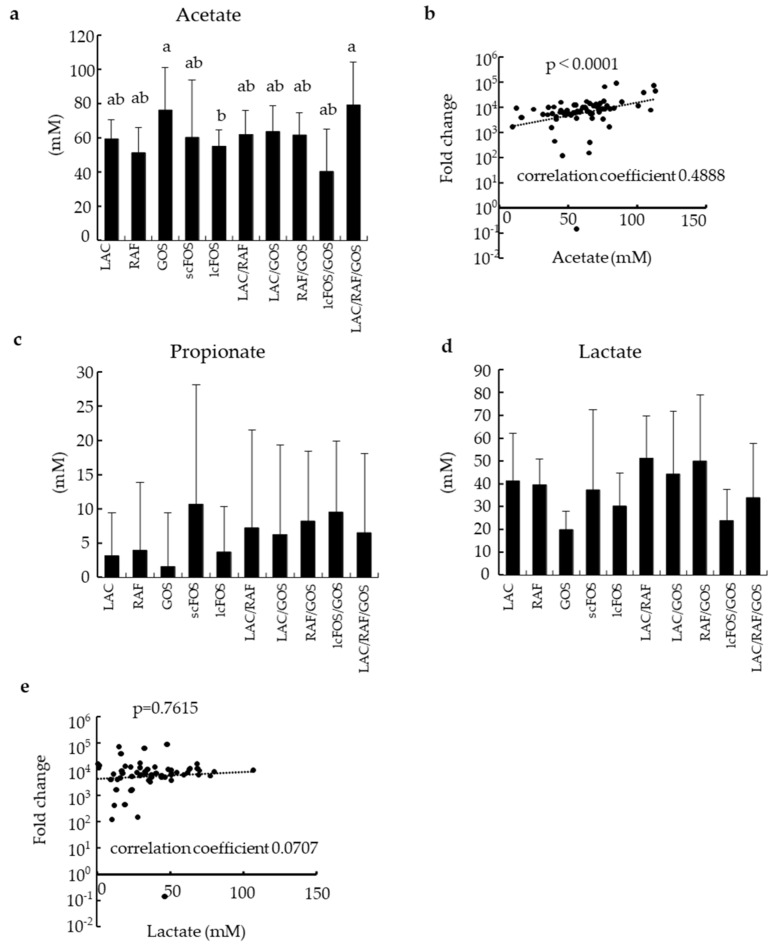
Analysis of SCFAs and lactate produced during cultivation. SCFAs and lactate produced during cultivation were calculated by subtracting the concentration at 0 h from that at 24 h. (**a**): acetate production, (**b**): correlation between the total number of *Bifidobacterium* and concentration of acetate produced, (**c**): propionate production, (**d**): lactate production, (**e**): correlation between the total number of *Bifidobacterium* and concentration of lactate produced. Data are expressed as means (n = 7) with SD. Different letters indicate significant differences (*p* < 0.05).

Acetate production was significantly higher in the GOS and LAC/RAF/GOS groups than in the other groups (Figure 2a) and correlated well with the growth of *Bifidobacterium* (Figure 2b). The concentrations of propionate and lactate are shown in Figure 2c and 2d, respectively. Although bifidobacteria are well-known producers of lactate, the production of lactate was not correlated with the growth of *Bifidobacterium* (Figure 2e). Butyrate was not detected in this study.

## 4. Discussion

The gut microbiota of breastfed infants is often dominated by bifidobacteria, especially infant-type HRB, compared to that of formula-fed infants. HMOs may be an underlying mechanism behind the selective promotion of infant-type HRB due to their selective prebiotic properties. As infant-type HRB are believed to contribute to infant health, it is a crucial developmental goal for infant formulas to exhibit HMO-like bifidogenic properties. As HMOs are a complex mixture of oligosaccharides with diverse structures, we hypothesized that combinations of alternative non-digestible oligosaccharides with structural diversity could replicate these bifidogenic properties. In this study, we identified an optimal combination of prebiotic oligosaccharides that effectively and evenly promotes the growth of all infant-type HRB, with LAC/RAF/GOS proving to be the most effective combination for supporting all tested infant-type HRB.

In accordance with previous findings [34], the colonization patterns of infant-type HRB in the gut microbiota were diverse among the infants in this study. Specifically, one to four species of infant-type HRB were detected, and four combinations of infant-type HRB species were observed among the seven infants. The carbohydrates utilized by *Bifidobacterium* are not uniform within the genus but vary at the species or even strain level [30,39,40]. Furthermore, processes of shaping infant-type *Bifidobacterium* communities are complicated, i.e., HRB species can affect each other’s dominance with ‘priority effects’, depending on carbohydrate utilization abilities [41], and cross-feedings of carbohydrates between species can occur [42,43]. Therefore, in this study, we presumed that it is difficult for a single oligosaccharide to promote the growth of all infant-type HRB species in all seven infants.

Our results demonstrated that no single oligosaccharide was able to evenly and effectively promote the growth of all infant-type HRB species in the tested infant feces. Specifically, lcFOS had limited effectiveness in promoting the growth of any infant-type HRB. LAC, RAF, and scFOS showed moderate and uneven prebiotic effects for all infant-type HRB. GOS could effectively promote the growth of *B. breve*, *B. longum* subsp. *infantis*, and *B. bifidum* but not that of *B. longum* subsp. *longum*.

Unexpectedly, lcFOS, a bifidogenic oligosaccharide commonly used for both adults and infants [44], had little effect on infant-type HRB in this study. Considering the cause of this, all subjects were under one year of age, and although some had started to consume solid foods, they generally had little feeding experience with fructans, which are often derived from vegetables and fruits. Furthermore, as of 2024, no infant formula containing lcFOS is available on the Japanese market; therefore, the subjects had never consumed lcFOS from infant formulas. Consequently, the intestinal environment of the subjects could not facilitate preferential colonization of bifidobacteria capable of utilizing lcFOS. Indeed, a previous report showed that changes in the gut microbiota due to lcFOS (inulin) intake are more pronounced in populations with high fiber intake than in populations with low fiber intake [45], suggesting that dietary habits influence responsiveness to prebiotics. Based on this idea, it is plausible that the high responsiveness of the subjects to GOS could be attributed to their dietary habits, that is, either exclusive or partial breastfeeding. Breast milk is rich in lactose, the building block of GOS, and contains GOS as well [46]. This may help shape the intestinal environment, which enriches the bifidobacteria of GOS-high utilizers. Taken together, we propose that the optimal prebiotic oligosaccharides for infants may be a combination that includes GOS, which could promote the growth of residential bifidobacteria that have been ‘primed’ to respond to GOS by breast milk. Future metagenomic analyses will help confirm this hypothesis by analyzing the presence of sugar utilization genes in the gut microbiota of infants.

Because GOS was unable to effectively promote the growth of *B. longum* subsp. *longum* in the subjects, an optimal combination would be a combination containing GOS that can improve this feature without compromising the advantages of GOS. From this perspective, the combination of LAC/RAF/GOS was not only the most effective in promoting the growth of *B. longum* subsp. *longum* in all subjects, but also demonstrated comparable or even better prebiotic effects on *B. breve*, *B. longum* subsp. *infantis*, and *B. bifidum* compared with GOS alone. We previously reported that the combination of LAC/RAF/GOS showed superior bifidogenic effects on *B. breve* and *B. longum* subsp. *longum* in a mixed culture system of seven bacterial strains [29]; however, it remained unclear whether the same effect was observed in every infant-type HRB and in the complex gut microbiota. In this study, LAC/RAF/GOS shows effective bifidogenic effects on all subjects’ infant-type HRB in the complex microbiota. These results suggest that LAC/RAF/GOS is a superior prebiotic mix for infants and is less likely to yield non-responders.

*B. longum* subsp. *longum* generally possesses a homolog of LT-SBP, one of the genes responsible for LAC utilization, and a homolog of RafB, one of the genes responsible for RAF utilization [30]. Additionally, LT-SBP is frequently found in *B. breve* and *B. bifidum*, whereas RafB is frequently found in *B. breve*, *B. longum* subsp. *infantis*, and *B. bifidum* [30]. These facts may partly explain why the combination of LAC/RAF/GOS showed superior effects, despite the GOS concentration being one-third that of the GOS alone group. However, considering that both LAC and RAF showed only weak to moderate bifidogenic effects on the HRB when used alone, this explanation may not be sufficient. Changes in the gene expression and metabolite profiles of infant-type HRB exposed to LAC/RAF/GOS should be analyzed in detail using transcriptomics and metabolomics.

In this study, we observed a positive correlation between the number of *Bifidobacterium* and acetate concentration, as well as tendency toward higher acetate production, in the LAC/RAF/GOS and GOS groups. Short-chain fatty acids produced by the gut microbiota, including acetate, have been reported to directly inhibit harmful bacteria [9] and act as signaling molecules contributing to host health by regulating immunity [47,48], metabolism [49], and endocrine functions [50]. In fact, an increase in the concentration of acetate in the colon and serum was observed in neonatal mice administered with the LAC/RAF/GOS [29]. In addition, enhanced immune and endocrine development has been observed, such as an increase in regulatory T cells in the colonic mucosal lamina propria and promotion of GLP-1 secretion [7]. Taking these findings into account, LAC/RAF/GOS is expected to contribute to health by promoting immune and endocrine development via acetate in human infants. However, further clinical studies are warranted. On the other hand, butyrate was not detected in this study, probably due to less proportion of butyrate-producing bacteria in the gut microbiota of infants who are continuously breastfed, as previously reported [51].

There are several limitations as follows. First, the sample size was rather small, i.e., only seven infants were included in this study, due to hardware limitations of the instrument used and the experimental difficulty in obtaining stable results. Second, the culture model in this study lacks host cells, so it cannot replicate the direct and indirect interactions between the host and gut microbiota, such as the modulation of the microbiota by the host immune system [47,52] or by antimicrobial peptides [53,54]. Third, the composition of the gut microbiota during infancy is strongly influenced by the defense molecules in breast milk, such as sIgA [55], lysozyme [56], and lactoferrin [57]. Fourth, actual effects of prebiotics are more complicated due to such as secondary effects from cross-feeding of prebiotics between different bacterial species [42,43]. The impact of cross-feedings on targeted infant-type HRB could not be evaluated in this study. Therefore, careful interpretation is required for secondary changes in the microbiota, beyond direct growth promotion and metabolite production by prebiotics, as this model cannot fully replicate in vivo responses. Further clinical trials are needed to verify whether the candidate of superior combination of prebiotic oligosaccharides found in this study actually yield fewer non-responders.

## 5. Conclusions

This is the first study to compare and evaluate the major prebiotic oligosaccharides used in infant formula in terms of lower yield of non-responders. We found that LAC/RAF/GOS is a superior combination supporting the growth of all infant-type HRB. Our findings may aid in the development of infant formulas that better align the gut microbiota of formula-fed infants with that of breastfed infants.

## Figures and Tables

**Table 1:** General information about the subjects.

Subject ID	1	2	3	4	5	6	7
Age (months) *	1.5	3	6.4	6.4	7.0	9.1	10.2
Breast-fed	+	+	+	+	+	+	+
Formula-fed			+		+	+	+
Solid food				+		+	+

* A fecal sample was collected from each subject at the indicated age. Feeding habits around a specific age are represented with the symbol “+”.

**Table 2:** Species of infant-type HRB in the gut microbiota of the subjects.

Subject ID	1	2	3	4	5	6	7
*B. breve*	+	+	+	+	+		+
*B. longum* subsp. *longum*		+		+	+	+	+
*B. longum* subsp. *infantis*		+		+	+	+	+
*B. bifidum*					+		+

Species detected by quantitative real-time PCR are indicated with the symbol “+”.

**Table 3:** Experimental groups.

	Groups									
	LAC	RAF	GOS	scFOS	lcFOS	LAC/ RAF	LAC/ GOS	RAF/ GOS	lcFOS/ GOS	LAC /RAF /GOS
Lactulose (LAC)	+					+	+			+
Raffinose (RAF)		+				+		+		+
Galactooligosaccharides (GOS)			+				+	+	+	+
Short-chain fructooligosaccharides (scFOS)				+						
Long-chain fructooligosaccharides (lcFOS)					+				+	

The prebiotics used in each group are indicated with the symbol “+”. The weight ratios of the prebiotics in combination were 1:1:1 (LAC/RAF/GOS), 1:1 (RAF/GOS, LAC/GOS, and LAC/RAF), and 1:9 (lcFOS/GOS). The total concentration of oligosaccharides (s) in each culture medium was 1.0% (*w*/*v*).

## Data Availability

The data used in this study can be found in the published article and its Supplementary Information.

## Supplementary Materials

The following supporting information can be downloaded at: https://www.mdpi.com/article/10.3390/nu16193347/s1, Figure S1: Analysis of the cell number of total bacteria; Figure S2: Analysis of the cell numbers of *Bifidobacterium* and each human-residential bifidobacteria (HRB) species. Table S1: PCR primers used to detect the bacterial species.

## References

[1] Saturio S., Nogacka A.M., Alvarado-Jasso G.M., Salazar N., de los Reyes-Gavilán C.G., Gueimonde M., Arboleya S. (2021). Role of Bifidobacteria on Infant Health. Microorganisms.

[2] Wong C.B., Sugahara H., Odamaki T., Xiao J.Z. (2018). Different Physiological Properties of Human-Residential and Non-Human-Residential Bifidobacteria in Human Health. Benef. Microbes.

[3] Sugahara H., Odamaki T., Hashikura N., Abe F., Xiao J.Z. (2015). Differences in Folate Production by Bifidobacteria of Different Origins. Biosci. Microbiota Food Health.

[4] Hiraku A., Nakata S., Murata M., Xu C., Mutoh N., Arai S., Odamaki T., Iwabuchi N., Tanaka M., Tsuno T. (2023). Early Probiotic Supplementation of Healthy Term Infants with Bifidobacterium Longum Subsp. Infantis M-63 Is Safe and Leads to the Development of Bifidobacterium-Predominant Gut Microbiota: A Double-Blind, Placebo-Controlled Trial. Nutrients.

[5] Wong C.B., Iwabuchi N., Xiao J.Z. (2019). Exploring the Science behind Bifidobacterium Breve M-16V in Infant Health. Nutrients.

[6] Henrick B.M., Rodriguez L., Lakshmikanth T., Pou C., Henckel E., Arzoomand A., Olin A., Wang J., Mikes J., Tan Z. (2021). Bifidobacteria-Mediated Immune System Imprinting Early in Life. Cell.

[7] Izumi H., Ehara T., Sugahara H., Matsubara T., Mitsuyama E., Nakazato Y., Tsuda M., Shimizu T., Odamaki T., Xiao J.Z. (2019). The Combination of Bifidobacterium Breve and Three Prebiotic Oligosaccharides Modifies Gut Immune and Endocrine Functions in Neonatal Mice. J. Nutr..

[8] Huda M.N., Ahmad S.M., Alam M.J., Khanam A., Kalanetra K.M., Taft D.H., Raqib R., Underwood M.A., Mills D.A., Stephensen C.B. (2019). Bifidobacterium Abundance in Early Infancy and Vaccine Response at 2 Years of Age. Pediatrics.

[9] Fukuda S., Toh H., Hase K., Oshima K., Nakanishi Y., Yoshimura K., Tobe T., Clarke J.M., Topping D.L., Suzuki T. (2011). Bifidobacteria Can Protect from Enteropathogenic Infection through Production of Acetate. Nature.

[10] Sakurai T., Odamaki T., Xiao J.Z. (2019). Production of Indole-3-Lactic Acid by Bifidobacterium Strains Isolated Fromhuman Infants. Microorganisms.

[11] Newburg D.S., Walker W.A. (2007). Protection of the Neonate by the Innate Immune System of Developing Gut and of Human Milk. Pediatr. Res..

[12] Lönnerdal B. (2003). Nutritional and Physiologic Significance of Human Milk Proteins. Am. J. Clin. Nutr..

[13] Lee S.A., Lim J.Y., Kim B.S., Cho S.J., Kim N.Y., Kim O.B., Kim Y. (2015). Comparison of the Gut Microbiota Profile in Breast-Fed and Formula-Fed Korean Infants Using Pyrosequencing. Nutr. Res. Pract..

[14] Sakanaka M., Gotoh A., Yoshida K., Odamaki T., Koguchi H., Xiao J.Z., Kitaoka M., Katayama T. (2020). Varied Pathways of Infant Gut-Associated Bifidobacterium to Assimilate Human Milk Oligosaccharides: Prevalence of the Gene Set and Its Correlation with Bifidobacteria-Rich Microbiota Formation. Nutrients.

[15] Soyyilmaz B., Mikš M.H., Röhrig C.H., Matwiejuk M., Meszaros-matwiejuk A., Vigsnæs L.K. (2021). The Mean of Milk: A Review of Human Milk Oligosaccharide Concentrations throughout Lactation. Nutrients.

[16] Yu Z.T., Chen C., Kling D.E., Liu B., McCoy J.M., Merighi M., Heidtman M., Newburg D.S. (2013). The Principal Fucosylated Oligosaccharides of Human Milk Exhibit Prebiotic Properties on Cultured Infant Microbiota. Glycobiology.

[17] Laursen M.F., Roager H.M. (2023). Human Milk Oligosaccharides Modify the Strength of Priority Effects in the Bifidobacterium Community Assembly during Infancy. ISME J..

[18] Schönknecht Y.B., Moreno Tovar M.V., Jensen S.R., Parschat K. (2023). Clinical Studies on the Supplementation of Manufactured Human Milk Oligosaccharides: A Systematic Review. Nutrients.

[19] Parschat K., Melsaether C., Jäpelt K.R., Jennewein S. (2021). Clinical Evaluation of 16-Week Supplementation with Tolerability, Safety and Effect on Growth. Nutrients.

[20] Bosheva M., Tokodi I., Krasnow A., Pedersen H.K., Lukjancenko O., Eklund A.C., Grathwohl D., Sprenger N., Berger B., Cercamondi C.I. (2022). Infant Formula with a Specific Blend of Five Human Milk Oligosaccharides Drives the Gut Microbiota Development and Improves Gut Maturation Markers: A Randomized Controlled Trial. Front. Nutr..

[21] Kiyosawa I., Takase M., Yamauchi K., Ono J., Yaeshima T., Okonogi S. (1986). Lactulose and Intestinal Microflora in Infant Nutrition. Bifidobact. Microflora.

[22] Nagendra R., Viswanatha S., Kumar S.A., Murthy B.K., Rao S.V. (1995). Effect of Feeding Milk Formula Containing Lactulose to Infants on Faecal Bifidobacterial Flora. Nutr. Res..

[23] Hattori K., Sasai M., Yamamoto A., Taniuchi S., Kojima T., Kobayashi Y., Iwamoto H., Yaeshima T., Hayasawa H. (2000). Intestinal Flora of Infants with Cow Milk Hypersensitivity Fed on Casein-Hydrolyzed Formula Supplemented Raffinose. Arerugi.

[24] Williams T., Choe Y., Price P., Katz G., Suarez F., Paule C., Mackey A. (2014). Tolerance of Formulas Containing Prebiotics in Healthy, Term Infants. J. Pediatr. Gastroenterol. Nutr..

[25] Paineau D., Respondek F., Menet V., Sauvage R., Bornet F., Wagner A. (2014). Effects of Short-Chain Fructooligosaccharides on Faecal Bifidobacteria and Specific Immune Response in Formula-Fed Term Infants: A Randomized, Double-Blind, Placebo-Cotrolled Trial. J. Nutr. Sci. Vitaminol..

[26] Veereman G. (2007). Pediatric Applications of Inulin and Oligofructose. J. Nutr..

[27] Ackerman D.L., Craft K.M., Townsend S.D. (2017). Infant Food Applications of Complex Carbohydrates: Structure, Synthesis, and Function. Carbohydr. Res..

[28] Scholtens P.A.M.J., Alliet P., Raes M., Alles M.S., Kroes H., Boehm G., Knippels L.M.J., Knol J., Vandenplas Y. (2008). Fecal Secretory Immunoglobulin A Is Increased in Healthy Infants Who Receive a Formula with Short-Chain Galacto-Oligosaccharides and Long-Chain Fructo-Oligosaccharides. J. Nutr..

[29] Ehara T., Izumi H., Tsuda M., Nakazato Y., Iwamoto H., Namba K., Takeda Y. (2016). Combinational Effects of Prebiotic Oligosaccharides on Bifidobacterial Growth and Host Gene Expression in a Simplified Mixed Culture Model and Neonatal Mice. Br. J. Nutr..

[30] Ojima M.N., Yoshida K., Sakanaka M., Jiang L., Odamaki T., Katayama T. (2022). Ecological and Molecular Perspectives on Responders and Non-Responders to Probiotics and Prebiotics. Curr. Opin. Biotechnol..

[31] Yoshida K., Hirano R., Sakai Y., Choi M., Sakanaka M., Kurihara S., Iino H., Xiao J.Z., Katayama T., Odamaki T. (2021). Bifidobacterium Response to Lactulose Ingestion in the Gut Relies on a Solute-Binding Protein-Dependent ABC Transporter. Commun. Biol..

[32] Satoh T., Odamaki T., Namura M., Shimizu T., Iwatsuki K., Nishimoto M., Kitaoka M., Xiao J. (2013). zhong In Vitro Comparative Evaluation of the Impact of Lacto-N-Biose I, a Major Building Block of Human Milk Oligosaccharides, on the Fecal Microbiota of Infants. Anaerobe.

[33] Murakami R., Hashikura N., Yoshida K., Xiao J.Z., Odamaki T. (2021). Growth-Promoting Effect of Alginate on Faecalibacterium Prausnitzii through Cross-Feeding with Bacteroides. Food Res. Int..

[34] Guo X., Xia X., Tang R., Zhou J., Zhao H., Wang K. (2008). Development of a Real-Time PCR Method for Firmicutes and Bacteroidetes in Faeces and Its Application to Quantify Intestinal Population of Obese and Lean Pigs. Lett. Appl. Microbiol..

[35] Matsuki T., Watanabe K., Fujimoto J., Takada T. (2004). Use of 16S rRNA Gene-Targeted Group-Specific Primers for Real-Time PCR Analysis of Predominant Bacteria in Human Feces. Appl. Environ. Microbiol..

[36] Matsuki T., Watanabe K., Tanaka R., Oyaizu H. (1998). Rapid Identification of Human Intestinal Bifidobacteria by 16S rRNA-Targeted Species- and Group-Specific Primers. FEMS Microbiol. Lett..

[37] Matsuki T., Watanabe K., Fujimoto J., Kado Y., Takada T., Matsumoto K., Tanaka R. (2004). Quantitative PCR with 16S rRNA-Gene-Targeted Species-Specific Primers for Analysis of Human Intestinal Bifidobacteria. Appl. Environ. Microbiol..

[38] Kato K., Odamaki T., Mitsuyama E., Sugahara H., Xiao J.Z., Osawa R. (2017). Age-Related Changes in the Composition of Gut Bifidobacterium Species. Curr. Microbiol..

[39] Xiao J.Z., Takahashi S., Nishimoto M., Odamaki T., Yaeshima T., Iwatsuki K., Kitaoka M. (2010). Distribution of In Vitro Fermentation Ability of Lacto-TV-Biose I, a Major Building Block of Human Milk Oligosaccharides, in Bifidobacteria! Strains. Appl. Environ. Microbiol..

[40] Watson D., O’Connell Motherway M., Schoterman M.H.C., van Neerven R.J.J., Nauta A., Van Sinderen D. (2013). Selective Carbohydrate Utilization by Lactobacilli and Bifidobacteria. J. Appl. Microbiol..

[41] Ojima M.N., Jiang L., Arzamasov A.A., Yoshida K., Odamaki T., Xiao J., Nakajima A., Kitaoka M., Hirose J., Urashima T. (2022). Priority Effects Shape the Structure of Infant-Type Bifidobacterium Communities on Human Milk Oligosaccharides. ISME J..

[42] Morozumi M., Wada Y., Tsuda M., Tabata F., Ehara T., Nakamura H., Miyaji K. (2023). Cross-Feeding among Bifidobacteria on Glycomacropeptide. J. Funct. Foods.

[43] Nishiyama K., Nagai A., Uribayashi K., Yamamoto Y., Mukai T., Okada N. (2018). Two Extracellular Sialidases from Bifidobacterium Bifidum Promote the Degradation of Sialyl-Oligosaccharides and Support the Growth of Bifidobacterium Breve. Anaerobe.

[44] Nagy D.U., Sándor-Bajusz K.A., Bódy B., Decsi T., Van Harsselaar J., Theis S., Lohner S. (2023). Effect of Chicory-Derived Inulin-Type Fructans on Abundance of Bifidobacterium and on Bowel Function: A Systematic Review with Meta-Analyses. Crit. Rev. Food Sci. Nutr..

[45] Healey G., Murphy R., Butts C., Brough L., Whelan K., Coad J. (2018). Habitual Dietary Fibre Intake Influences Gut Microbiota Response to an Inulin-Type Fructan Prebiotic: A Randomised, Double-Blind, Placebo-Controlled, Cross-over, Human Intervention Study. Br. J. Nutr..

[46] Sumiyoshi W., Urashima T., Nakamura T., Arai I., Nagasawa T., Saito T., Tsumura N., Wang B., Brand-Miller J., Watanabe Y. (2004). Galactosyllactoses in the Milk of Japanese Women: Changes in Concentration during the Course of Lactation. J. Appl. Glycosci..

[47] Takeuchi T., Miyauchi E., Kanaya T., Kato T., Nakanishi Y., Watanabe T., Kitami T., Taida T., Sasaki T., Negishi H. (2021). Acetate Differentially Regulates IgA Reactivity to Commensal Bacteria. Nature.

[48] Antunes K.H., Fachi J.L., de Paula R., da Silva E.F., Pral L.P., dos Santos A.Á., Dias G.B.M., Vargas J.E., Puga R., Mayer F.Q. (2019). Microbiota-Derived Acetate Protects against Respiratory Syncytial Virus Infection through a GPR43-Type 1 Interferon Response. Nat. Commun..

[49] Kimura I., Ozawa K., Inoue D., Imamura T., Kimura K., Maeda T., Terasawa K., Kashihara D., Hirano K., Tani T. (2013). The Gut Microbiota Suppresses Insulin-Mediated Fat Accumulation via the Short-Chain Fatty Acid Receptor GPR43. Nat. Commun..

[50] Wichmann A., Allahyar A., Greiner T.U., Plovier H., Lundén G.Ö., Larsson T., Drucker D.J., Delzenne N.M., Cani P.D., Bäckhed F. (2013). Microbial Modulation of Energy Availability in the Colon Regulates Intestinal Transit. Cell Host Microbe.

[51] Tsukuda N., Yahagi K., Hara T., Watanabe Y., Matsumoto H., Mori H., Higashi K., Tsuji H., Matsumoto S., Kurokawa K. (2021). Key Bacterial Taxa and Metabolic Pathways Affecting Gut Short-Chain Fatty Acid Profiles in Early Life. ISME J..

[52] Weis A.M., Round J.L. (2021). Microbiota-Antibody Interactions That Regulate Gut Homeostasis. Cell Host Microbe.

[53] Masuda K., Nakamura K., Yoshioka S., Fukaya R., Sakai N., Ayabe T. (2011). Regulation of Microbiota by Antimicrobial Peptides in the Gut. Adv. Otorhinolaryngol..

[54] Ostaff M.J., Stange E.F., Wehkamp J. (2013). Antimicrobial Peptides and Gut Microbiota in Homeostasis and Pathology. EMBO Mol. Med..

[55] Donald K., Petersen C., Turvey S.E., Finlay B.B., Azad M.B. (2022). Review Secretory IgA: Linking Microbes, Maternal Health, and Infant Health through Human Milk. Cell Host Microbe.

[56] Minami J., Odamaki T., Hashikura N., Abe F., Xiao J.Z. (2016). Lysozyme in Breast Milk Is a Selection Factor for Bifidobacterial Colonisation in the Infant Intestine. Benef. Microbes.

[57] Gopalakrishna K.P., Hand T.W. (2020). Influence of Maternal Milk on the Neonatal Intestinal Microbiome. Nutrients.

