# Supplementary material for: Comparative Study of Prebiotics for Infants Using a Fecal Culture System: Insights into Responders and Non-Responders
Source: Nutrients. 2024 Oct 2;16(19):3347. doi: 10.3390/nu16193347 (PMC11478422; doi:10.3390/nu16193347)
Supplement: Supplementary file 1 [file nutrients-16-03347-s001.zip › nutrients-3237832-supplementary.pdf]

nutrients

MDPI

Supplemental Table S1. PCR primers used to detect the bacterial species

Target	Primer	Sequences (5'-3')	Annealing temperature	Reference
Total bacteria	Eub338F	ACTCCTACG GGAGGCAGCAG	60 °C	[34]
	Eub518R	ATTACCGCG GCTGCTGG		
Bifidobacterium	g-Bifid-F	CTCCTGGAAACGGGTGG	55 °C	[35]
	g-Bifid-R	GGTGTTCTTCCCGATATCTACA		
Bifidobacterium bifidum	BiBIF-1	CCACATGATCGCATGTGATTG	55 °C	[36]
	BiBIF-2	CCGAAGGCTTGCTCCCAAA		
Bifidobacterium breve	BiBRE-1	CCGGATGCTCCATCACAC	55 °C	[37]
	BiBRE-2	ACAAAGTGCCTTGCTCCCT		
Bifidobacterium longum subsp. *longum*	BiLON-1	TTCCAGTTGATCGCATGGTC	55 °C	[37]
	BiLON-2	GGGAAGCCGTATCTCTACGA		
Bifidobacterium longum subsp. *infantis*	BiINF-1	TTCCAGTTGATCGCATGGTC	55 °C	[37]
	BiINF-2	GGAAACCCCATCTCTGGGAT		

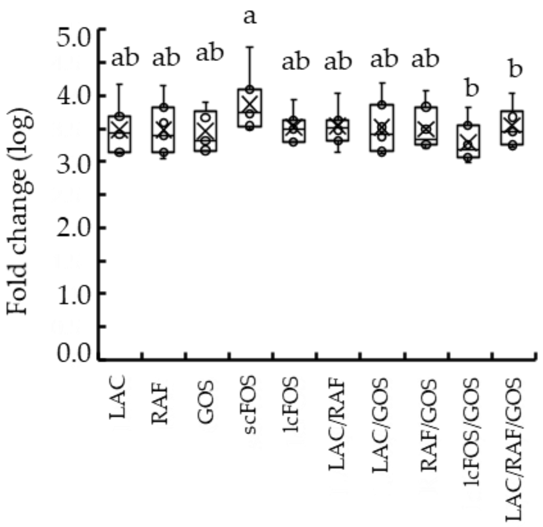

Supplemental Figure S1.

Analysis of the cell number of total bacteria. Log-fold changes in the cell number of total bacteria from 0 h to 24 h. Data are expressed as means (n=7) with SD. Different letters indicate significant differences ($P < 0.05$)

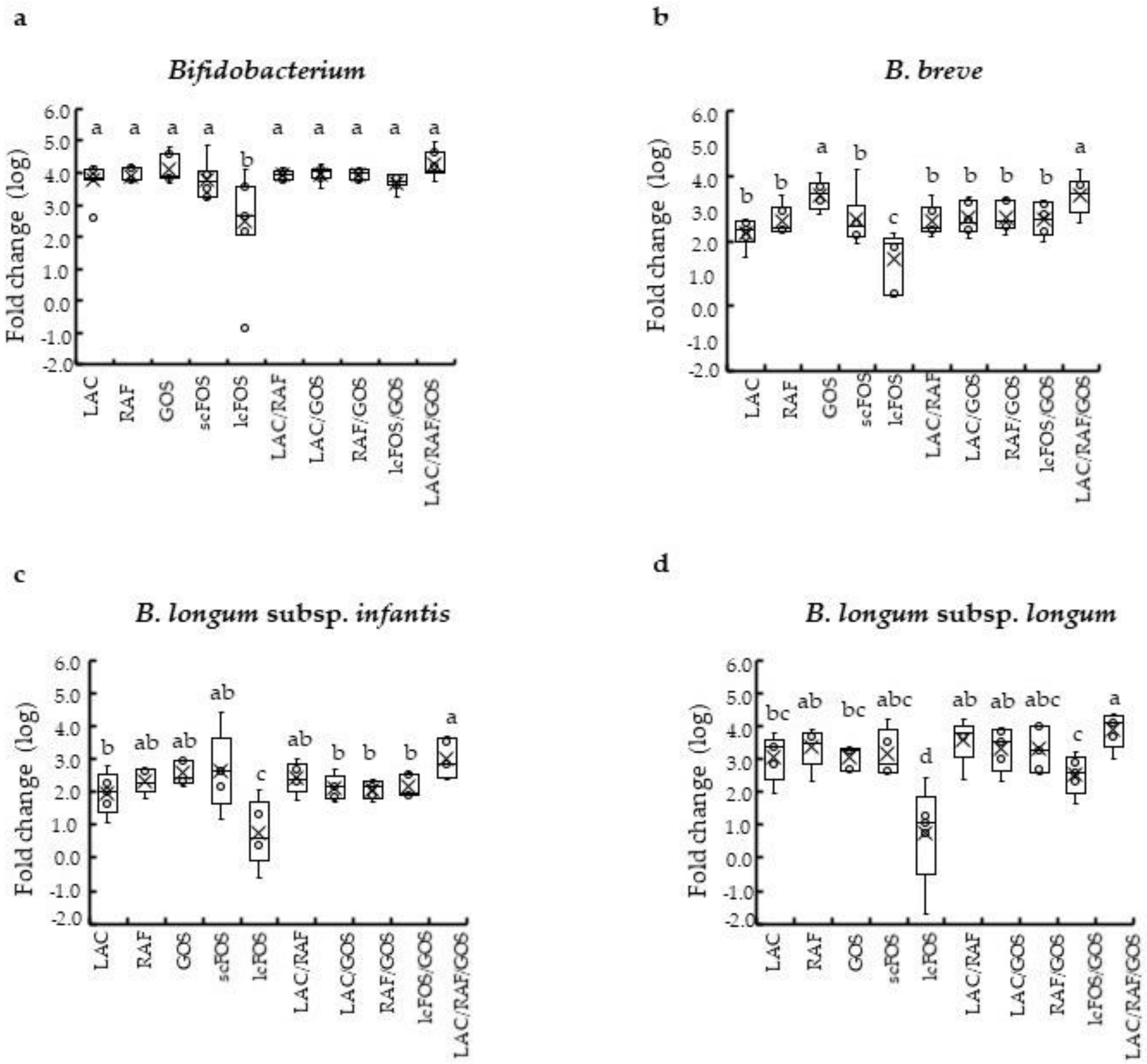

Supplemental Figure S2.

Analysis of the cell numbers of *Bifidobacterium* and each human-residential bifidobacteria (HRB) species. Log-fold changes in the cell numbers of targeted bifidobacteria from 0 h to 24 h. a: *Bifidobacterium*, b: *Bifidobacterium breve*, c: *Bifidobacterium longum* subsp. *infantis*, d: *Bifidobacterium longum* subsp. *longum*. Data are expressed as means (n=7) with SD. Different letters indicate significant differences ($P < 0.05$).
